# Hospital and Neurodevelopmental Outcomes in Nano-Preterm Infants Receiving Invasive vs Noninvasive Ventilation at Birth

**DOI:** 10.1001/jamanetworkopen.2022.29105

**Published:** 2022-08-29

**Authors:** Vivek V. Shukla, J. Paige Souder, Grant Imbrock, Muhan Hu, A. K. M. Fazlur Rahman, Colm P. Travers, Namasivayam Ambalavanan, Waldemar A. Carlo, Charitharth Vivek Lal

**Affiliations:** 1 Division of Neonatology, Department of Pediatrics, University of Alabama at Birmingham, Birmingham; 2 School of Medicine, University of Alabama at Birmingham, Birmingham; 3 Department of Biostatistics, University of Alabama at Birmingham, Birmingham

## Abstract

**Importance:**

Infants with gestational age between 22 0/7 and 23 6/7 weeks (referred to as nano-preterm infants) are at very high risk of adverse outcomes. Noninvasive respiratory support at birth improves outcomes in infants born at 24 0/7 to 27 6/7 weeks’ gestational age. Evidence is limited on whether similar benefits of non-invasive respiratory support at birth extend to nano-preterm infants.

**Objective:**

To evaluate the hypothesis that intubation at 10 minutes or earlier after birth is associated with a higher incidence of bronchopulmonary dysplasia (BPD) or death by 36 weeks’ postmenstrual age (PMA) in nano-preterm infants.

**Design, Setting, and Participants:**

This observational cohort study included all nano-preterm infants at a level IV neonatal intensive care unit who were delivered from January 1, 2014, to June 30, 2021. Infants receiving palliative or comfort care at birth were excluded.

**Exposures:**

Infants were grouped based on first intubation attempt timing after birth (>10 minutes after birth and ≤10 minutes as noninvasive and invasive respiratory support at birth groups, respectively).

**Main Outcomes and Measures:**

The primary outcome was the composite outcome of BPD (physiological definition) or death by 36 weeks’ PMA.

**Results:**

All 230 consecutively born, eligible nano-preterm infants were included, of whom 88 (median [IQR] gestational age, 23.6 [23.4-23.7] weeks; 45 [51.1%] female; 54 [62.1%] Black) were in the noninvasive respiratory support at birth group and 142 (median [IQR] gestational age, 23.0 [22.4-23.3] weeks; 71 [50.0%] female; 94 [66.2%] Black) were in the invasive respiratory support at birth group. The incidence of BPD or death by 36 weeks’ PMA did not differ between the noninvasive and invasive respiratory support groups (83 of 88 [94.3%] in the noninvasive group vs 129 of 142 [90.9%] in the invasive group; adjusted odds ratio, 2.09; 95% CI, 0.60-7.25; *P* = .24). Severe intraventricular hemorrhage or death by 36 weeks’ PMA was lower in the invasive respiratory support at birth group (adjusted odds ratio, 2.20; 95% CI, 1.07-4.51; *P* = .03).

**Conclusions and Relevance:**

This cohort study’s findings suggest that noninvasive respiratory support in the first 10 minutes after birth is feasible but is not associated with a decrease in the risk of BPD or death compared with intubation and early surfactant delivery in nano-preterm infants.

## Key Points

**Question:**

Is intubation at 10 minutes or less after birth associated with a higher incidence of bronchopulmonary dysplasia or death by 36 weeks’ postmenstrual age in nano-preterm (22 0/7 to 23 6/7 weeks’ gestational age) infants?

**Findings:**

This cohort study of 230 nano-preterm infants found that the incidence of bronchopulmonary dysplasia or death by 36 weeks’ postmenstrual age did not significantly differ between those exposed to noninvasive and invasive respiratory support at birth. Noninvasive respiratory support at birth may be associated with a higher risk of severe intraventricular hemorrhage or death by 36 weeks’ postmenstrual age in nano-preterm infants.

**Meaning:**

These findings suggest that noninvasive respiratory support in the first 10 minutes after birth is feasible in the treatment of nano-preterm infants, although it may be associated with an increased incidence of severe intraventricular hemorrhage or death by 36 weeks’ postmenstrual age.

## Teaser

This cohort study of nano-preterm infants examines whether early intubation after birth is associated with a higher incidence of bronchopulmonary dysplasia or death.

## Introduction

Advances in neonatal-perinatal medicine have improved the survival of infants with progressively lower gestational age during the last few decades.^1^ Infants born between 22 0/7 and 23 6/7 weeks’ gestational age are considerably more immature and at a much higher risk of mortality and morbidity than infants born between 24 0/7 and 27 6/7 weeks’ gestational age.^1,2^ Several interventional trials^3,4,5^ have identified treatments to improve survival and outcomes of infants between 24 0/7 and 27 6/7 weeks’ gestational age. Bronchopulmonary dysplasia (BPD) is one of the leading morbidities in nano-preterm infants, and it is even higher in infants between 22 0/7 and 23 6/7 weeks’ gestational age. Noninvasive respiratory support at birth is now a commonly practiced intervention in infants born between 24 0/7 and 27 6/7 weeks’ gestation age because several large randomized clinical trials, such as the COIN (Continuous Positive Airway Pressure or Intubation at Birth) trial,^3^ SUPPORT (Surfactant, Positive Pressure, and Oxygenation Randomized Trial),^4^ and the Vermont Oxford Network (VON) trial,^5^ have shown that noninvasive respiratory support at birth improves respiratory outcomes in nano-preterm infants.

Infants born between 22 0/7 and 23 6/7 weeks’ gestational age are a highly specialized niche subgroup within the extremely preterm infant population (those born at <27 6/7 weeks’ gestational age) for which a proper descriptive term does not exist. Through this article, we propose the term nano-preterm for infants born between the gestational ages of 22 0/7 and 23 6/7 weeks to differentiate and precisely represent this population. Strong evidence is lacking on whether noninvasive respiratory support at birth is beneficial in nano-preterm infants. The current study aims to address this knowledge gap and evaluate the hypothesis that intubation at 10 minutes or earlier after birth is associated with a higher incidence of BPD or death by 36 weeks’ postmenstrual age (PMA) in nano-preterm infants. The intubation after 10 minutes was chosen as a surrogate for an attempted trial of noninvasive respiratory support after birth.

## Methods

### Study Design and Participants

The current study is a retrospective cohort study of actively treated nano-preterm infants born at the University of Alabama at Birmingham from January 1, 2014, to June 31, 2021, and cared for in the level IV neonatal intensive care unit. Infants receiving palliative or comfort care at birth were excluded. Data were abstracted from a research database of data prospectively collected by trained research nurses, and the outcomes were confirmed by reviewing electronic medical records. Race and ethnicity classification was based on self-reported maternal race and ethnicity. Data on sex, race, and ethnicity were collected to compare baseline characteristic distribution between the study groups. Infants were grouped based on first intubation attempt timing after birth (>10 minutes after birth and ≤10 minutes as noninvasive and invasive respiratory support at birth groups, respectively). The institutional review board at the University of Alabama at Birmingham approved the study with a waiver of informed consent because of the noninterventional and observational nature of the study. This study followed the Strengthening the Reporting of Observational Studies in Epidemiology (STROBE) reporting guideline.^6^

### Outcomes

Because death is a competing outcome for prematurity-related morbidities, the composite outcome of prematurity-related morbidities or death was used. The primary outcome was the composite outcome of BPD (physiological definition)^7^ or death by 36 weeks’ PMA. Major prematurity-related short- and long-term morbidities were included as secondary outcomes. Secondary outcomes included hemodynamically significant patent ductus arteriosus,^8^ necrotizing enterocolitis (modified Bell stage ≥2),^9^ severe intraventricular hemorrhage (grade 3 or 4),^10^ retinopathy of prematurity (stage ≥2),^11^ and moderate and/or severe neurodevelopmental disability at 22 to 30 months.^12^ Short-term secondary outcomes were assessed as a composite outcome with death by 36 weeks’ PMA, and long-term outcomes were assessed as a composite outcome with death by 22 to 30 months.

### Statistical Analysis

Descriptive analyses (means [SDs], medians [IQRs], and frequency distributions) were used to describe baseline characteristics stratified by groups. The χ^2^, Fisher exact, Wilcoxon rank sum, and 2-tailed, unpaired *t* tests as appropriate were used to compare characteristics between groups. Multivariable logistic regression analyses were performed with adjustment for previously reported risk factors associated with death and neurodevelopmental disability, including antenatal corticosteroids, birth weight, and gestational age,^13^ to quantify the adjusted odds ratios (aOR) and the 95% CIs for the association of timing of intubation by the study group on the outcomes. Analysis was performed using Stata statistical software, version 16 (StataCorp LLC) and SAS statistical software, version 9.4 (SAS Institute Inc). A 2-sided *P* < .05 was considered to be statistically significant.

## Results

All 230 consecutively born, eligible nano-preterm infants were included, of whom 88 were in the noninvasive respiratory support at birth group (45 [51.1%] female and 43 [48.9%] male; 54 [62.1%] Black, 4 [4.6%] Hispanic, and 29 [33.3%] White) and 142 in the invasive respiratory support at birth group (71 [50.0%] female and 71 [50.0%] male; 94 [66.2%] Black, 3 [2.1%] Hispanic, and 42 [29.6%] White). Gestational age (median, 23.6 weeks [IQR, 23.4-23.7 weeks] vs 23.0 weeks [IQR, 22.4-23.3 weeks]; *P* < .001) and birth weight (mean [SD], 577 [102] g vs 522 [82] g; *P* < .001) were higher in the noninvasive respiratory support at birth group vs the invasive group. Forty-nine of the 88 infants (55.7%) in the noninvasive respiratory support at birth group were later intubated. The timing of intubation was within the first hour after birth for most infants who required intubation in the noninvasive respiratory support at birth group (38 in the first hour, 4 in the second hour, 5 in 2-24 hours, and 2 on the second day). Of the 230 infants, 204 were born 22 months before June 2021 and were eligible for 22- to 30-month outcome assessment; 192 infants had 22- to 30-month outcomes available, and 12 infants were unavailable for follow-up. The noninvasive respiratory support at birth group had higher gestational age, higher birth weight, and more antenatal corticosteroid exposure (Table 1).

**Table 1.** Baseline Characteristics by Group

Characteristic	Noninvasive respiratory support at birth (n = 88)^a^	Invasive respiratory support at birth (n = 142)^b^	*P* value
Sex, No. (%)			
Female	45 (51.1)	71 (50.0)	.86
Male	43 (48.9)	71 (50.0)	
Gestational age, median (IQR), wk	23.6 (23.4-23.7)	23.0 (22.4-23.3)	<.001
Birth weight, mean (SD), g	577 (102)	522 (82)	<.001
Race and ethnicity, No. (%)			
Black	54 (62.1)	94 (66.2)	.37
Hispanic	4 (4.6)	3 (2.1)	
White	29 (33.3)	42 (29.6)	
Use of antenatal corticosteroids, No. (%)	77 (87.5)	108 (76.1)	.03

^a^ First intubation attempt at more than 10 minutes after birth.

^b^ First intubation attempt at 10 minutes or earlier after birth.

The incidence of BPD or death by 36 weeks’ PMA was 94.3% (83 of 88 patients) in the noninvasive respiratory support at birth group and 90.9% (129 of 142 patients) in the invasive respiratory support at birth group. On multivariable logistic regression analyses, no significant difference was found for BPD or death by 36 weeks’ PMA (aOR, 2.09; 95% CI, 0.60-7.25; *P* = .24) between the groups. Severe intraventricular hemorrhage or death by 36 weeks’ PMA was lower in the invasive respiratory support at birth group (aOR, 2.20; 95% CI, 1.07-4.51; *P* = .03). All other secondary outcomes were not significantly different (Table 2).

**Table 2.** Outcome Analyses by Group

Outcome	No./total No. (%)		Unadjusted		Adjusted	
	Noninvasive respiratory support at birth^a^	Invasive respiratory support at birth^b^	Odds ratio (95% CI)	*P* value	Odds ratio (95% CI)^c^	*P* value
BPD or death by 36 weeks’ PMA	83/88 (94.3)	129/142 (90.9)	1.63 (0.57-4.86)	.35	2.09 (0.60-7.25)	.24
Symptomatic PDA or death by 36 weeks’ PMA	42/88 (47.7)	58/142 (40.9)	1.32 (0.74-2.26)	.31	1.61 (0.86-3.02)	.14
NEC (Bell stage ≥2) or death by 36 weeks’ PMA	46/88 (52.3)	85/142 (59.9)	0.73 (0.43-1.25)	.26	1.84 (0.91-3.72)	.09
Severe IVH (grade 3 or 4) or death by 36 weeks’ PMA	52/88 (59.1)	92/142 (64.8)	0.78 (0.45-1.35)	.39	2.20 (1.07-4.51)	.03
ROP (stage ≥2) or death by 36 weeks’ PMA	80/88 (90.9)	123/142 (86.6)	1.54 (0.64-3.70)	.33	2.30 (0.83-6.33)	.11
Moderate or severe disability or death by 22-30 mo	47/70 (67.1)	100/122 (82.0)	0.45 (0.23-0.89)	.02	1.07 (0.44-2.60)	.87
Severe disability or death by 22-30 mo	46/70 (65.7)	99/122 (81.2)	0.44 (0.23-0.87)	.02	1.09 (0.45-2.61)	.84

Abbreviations: BPD, bronchopulmonary dysplasia; IVH, intraventricular hemorrhage; NEC, necrotizing enterocolitis; PDA, patent ductus arteriosus; PMA, postmenstrual age; ROP, retinopathy of prematurity.

^a^ First intubation attempt at more than 10 minutes after birth.

^b^ First intubation attempt at 10 minutes or earlier after birth.

^c^ Adjusted for antenatal corticosteroids, birth weight, gestational age by logistic regression analyses. The adjusted odds ratios represented in the table are for noninvasive respiratory support at birth group.

## Discussion

This study aimed to evaluate the association between invasive respiratory support at birth and BPD or death by 36 weeks’ PMA in nano-preterm infants. The study results suggest that noninvasive respiratory support in the first 10 minutes after birth is feasible but is not associated with a decrease in the risk of BPD or death compared with intubation and early surfactant delivery in nano-preterm infants.

To our knowledge, no comparative studies have been performed on the outcomes of noninvasive respiratory support in nano-preterm infants. Trials with infants of more than 24 weeks’ gestational age have identified noninvasive respiratory support as beneficial. The COIN trial,^3^ SUPPORT,^4^ and the VON trial^5^ have shown improvements in short-term respiratory outcomes, but the differences between the groups for BPD or death at 36 weeks were not statistically significant. No significant difference between invasive and noninvasive respiratory support for the composite outcome of mortality or neurodevelopmental disability was reported in SUPPORT,^14^ whereas the COIN and VON trials have not reported neurodevelopmental outcomes. Given the benefits in short-term respiratory outcomes, noninvasive respiratory support at birth has been established as the standard of care in infants between 24 0/7 and 27 6/7 weeks’ gestational age at birth. A trial of less invasive surfactant delivery that included infants with gestational ages between 23 0/7 and 26 6/7 weeks has also been published^15^; however, this trial had very few infants born at 23 weeks’ gestational age (19 of 211 [10%]), and gestational age–specific outcome analyses were not reported.

### Limitations

This study has several limitations. The study was a retrospective, single-center study. The baseline differences between the study groups favored the infants in the noninvasive respiratory support at birth group. The cohort design may also favor the noninvasive respiratory support at birth group because the infants did not need endotracheal intubation in the first 10 minutes after birth. The intended treatment for infants intubated in the first 10 minutes might have been different from the study group assignment based on the timing of intubation. Furthermore, most of the outcomes did not differ between groups.

## Conclusions

The findings of this cohort study suggest that noninvasive respiratory support in the first 10 minutes after birth is feasible but is not associated with a decrease in the risk of BPD or death compared with intubation and early surfactant delivery in nano-preterm infants. An adequately powered trial is needed to confirm the trends for better short-term outcomes in the invasive respiratory support at birth group seen in the current study.
